# Comparison of the uptake of health assessment items for Aboriginal and Torres Strait Islander people and other Australians: Implications for policy

**DOI:** 10.1186/1743-8462-2-21

**Published:** 2005-09-09

**Authors:** Margaret Kelaher, David Dunt, David Thomas, Ian Anderson

**Affiliations:** 1 Program Evaluation Unit, School of Population Health, University of Melbourne, Australia; 2 Onemda VicHealth Koori Health Unit, School of Population Health, University of Melbourne, Australia; 3 Centre for Health in Society, School of Population Health, University of Melbourne, Australia

## Abstract

**Background:**

Health Assessment (HA) items were introduced in 1999 for Aboriginal and Torres Strait Islander people aged at least 55 years and all Australians aged over 75 years. In 2004 a new item was introduced for HAs among adult Aboriginal and Torres Strait Islander people aged 15–54 years. The new item has been applauded as a major policy innovation however this enthusiasm has been tempered with concern about potential barriers to its uptake. In this study we aim to determine whether there are disparities in uptake of HA items for Aboriginal and Torres Strait Islander people compared to other Australians.

**Method:**

The analysis was based on Health Insurance Commission data. Indigenous status was ascertained based on the item number used. Logistic regression was used to compare uptake of HA items for older people among Aboriginal and Torres Strait Islander people compared to other Australians. Adjustments were made for dual eligibility. Uptake of the HA items for older people was compared to the uptake of the new item for Aboriginal and Torres Strait Islander people aged 15–44 years.

**Results:**

Our analyses suggest a significant and persistent disparity in the uptake of items for older patients among Aboriginal and Torres Strait Islander people compared to other Australians. A similar disparity appears to exist in the uptake of the new adult Aboriginal and Torres Strait Islander HA item.

**Conclusion:**

Further engagement of primary care providers and the community around the uptake of the new HA items may be required to ensure that the anticipated health benefits eventuate.

## 

The introduction of Medical Benefits Scheme (MBS) item numbers to reimburse health assessments (HAs) represented a major shift in support for access to health promotion and preventive care in primary care settings. The HA items provide reimbursement for doctors to evaluate patient's physical, psychological and social function in order to optimise health care and education. HA items were first introduced for older patients in 1999. [1] The items included HAs conducted at consulting rooms and not at consulting rooms, hospitals or residential aged care facilities (referred to hereafter as non-consulting room items). [1] Aboriginal and Torres Strait Islander people aged at least 55 years and all Australians aged over 75 years are eligible for these items. The item numbers for Aboriginal and Torres Strait Islander people and all Australians are shown in table 1.

**Table 1:** MBS Health Assessment item numbers

Health Assessment	All Australians 75+ yrs	Aboriginal and Torres Strait Islander people 55+yrs
At consulting room	700	704
Not at consulting rooms, hospitals or residential aged care facilities	702	706

The uptake of the HA items and other items introduced as part of the Enhanced Primary Care (EPC) program from 1999–2001 has been rigorously evaluated. HA items had the highest uptake of the Enhanced Primary Care items with around 18% of the eligible population using them. [2,3] No information was available on baseline levels for the provision of HAs but the evaluation did suggest that there was an increase in the use of HAs in case study practices and that reimbursement was an incentive to completing HAs in about one third of practices. Health benefits associated with HA among older patients were relatively small [4,5] and the evaluation suggested that further uptake was required to have significant impact on the health of the target populations. [3] This was particularly true of the items for Aboriginal and Torres Strait Islander people which were used at a significantly lower rate than the items for the general population. [6] It was suggested that this effect may have occurred either because Aboriginal and Torres Strait Islander people might be more likely to have pre-existing care plans or because Aboriginal and Torres Strait Islander people were more likely to use services (e.g. hospitals) where Medicare was not used. [7] In either case it would be expected that the disparity should decrease over time as people required new health assessments and Medicare use among indigenous people increased. [8]

In May 2004, a new item (item 710) was introduced for HAs among adult Aboriginal and Torres Strait Islander people aged 15–54 years. [8,9] Adult HAs could have significant health benefits for indigenous people because of the early age of onset of chronic disease and higher rates of infectious disease in this community compared to other Australians. [10] For example, the rate of sexually transmitted infection was halved at two year follow-up in indigenous rural and remote communities in Queensland where Well Persons Health Checks were conducted. [11] If the new item results in increased HAs, it has the potential to greatly reduce the burden of disease among indigenous Australians; it has rightly been applauded as an example of innovative policy in indigenous health. [9] However this enthusiasm has been tempered with concerns that the potential health benefits of the new item will not be realised because of low uptake. [9]

In this study we aim to establish whether there are likely to be barriers to the uptake of the new HA item by comparing the uptake of the HA items for older people among Aboriginal and Torres Strait Islander people and the rest of the community. We also examine differences in uptake over time and differences between States and Territories. Finally we compare uptake of the HA items for older people to the uptake of the new items for Aboriginal and Torres Strait Islander people aged 15–44 years in the first three quarters after their introduction. It would be expected that structural barriers to the introduction of HAs should have decreased over since 1999 because of the introduction of the HA items. Accordingly it might be expected that the uptake of the new item might be more rapid than the uptake of the items for older Australians.

### Data

Data on the use of item numbers (700, 702, 704, 706) by year and by State and Territory were obtained from the Health Insurance Commission statistical reports. [12] Data on the HA items was available from the last quarter in 1999 but this was not used in the general comparison because a full years data was not available. The extract included annual data from 2000–2004.

Data on the use of item numbers (700, 704, 710) in the first three quarters of their introduction was also obtained from the Health Insurance Commission statistical reports. [12] These data are available by State and Territory but figures for the whole of Australia were used because of low numbers. For items 702 and 704 the first three quarters data was for the last quarter of 1999 and the first two quarters of 2000. For item 710 the data was from the last three quarters of 2004. It should be noted that the first quarter data may not include data for the whole quarter.

In addition to the other eligibility requirements, only one claim could be made per person in a 12 month period. Accordingly quarterly and annual data reports should only contain one observation per person. Data are available for smaller geographic areas than State and Territory, such as general practice divisions, however low numbers and a high level of suppressed data made small area analysis problematic.

Population estimates for the Aboriginal and Torres Strait Islander population aged at least 55 years and aged 15–44 years by State and Territory were obtained for the Australian Bureau of Statistics (ABS) projections from the 2001 census for the years 2001 to 2004. [13] Population projections for the years 1999 and 2000 were obtained from series developed from the 1996 census. [14] The projections provide a low and high series of population estimates. In this study the series used had little impact on the results. The low series is reported because it yields the most conservative estimates of the difference between Aboriginal and Torres Strait Islander people and the rest of the community. Population estimates for the general population aged at least 75 years were obtained using ABS time series data. [15]

### Analysis

A logistic regression was conducted to analyse differences in the uptake of consulting room (700, 704) and non-consulting room (702, 706) HA items according to Indigenous status and year taking into account variation due to State and Territory. Consulting room and non-consulting room items were analysed separately because there is geographic variation in their use which may be potential source of confounding. The dependent variable was coded dichotomously using service use data to estimate the number of people who used the service and population data to estimate the number of people who did not. Year was coded to enable linear trends in uptake to be tested. Indigenous status was coded dichotomously based on whether the items were only available to Aboriginal and Torres Strait Islander people or available to all Australians.

The 12.2% of Aboriginal and Torres Strait Islander people aged at least 75 years would be eligible for the general population items as well as the Aboriginal and Torres Strait Islander specific items. All analyses were conducted twice to explore whether dual eligibility could have an impact on the results. The first set of analyses was based on observed service use. Service use among Aboriginal and Torres Strait Islander people would be underestimated in these analyses if people with dual eligibility were using general population items. The data were also analysed assuming that Aboriginal and Torres Strait Islander people aged at least 75 years accessed HAs through general population items at the same rate as the rest of community. These instances of service use were then attributed to Aboriginal and Torres Strait Islander people rather than to other Australians. Service use among Aboriginal and Torres Strait Islander people would be overestimated in these analyses because some of the people using the Aboriginal and Torres Strait Islander items are likely to be aged at least 75 years and therefore would be counted twice. Some overestimation would also be expected to occur because the observed rate of service use among Aboriginal and Torres Strait Islander people aged over 75 years is likely to be less than that for the general population.

Differences in rates of consulting room and non-consulting room service use for Aboriginal and Torres Strait Islander people and the rest of the community were calculated for each State and Territory.

A logistic regression was conducted to compare the uptake of older all Australian (700), older Aboriginal and Torres Strait Islander (704) and adult Aboriginal and Torres Strait Islander people (710) HA items. The HA item for adult Aboriginal and Torres Strait Islander people (710) was used as the reference category for comparisons. Quarter was coded to enable linear and quadratic trends in uptake to be tested. The dependent variable was coded dichotomously using service use data to estimate the number of people who used the service and population data to estimate the number of people who did not.

## Results

### Comparison of the uptake of HA items for older people among Aboriginal and Torres Strait Islander people and other Australians

**Figure 1:**
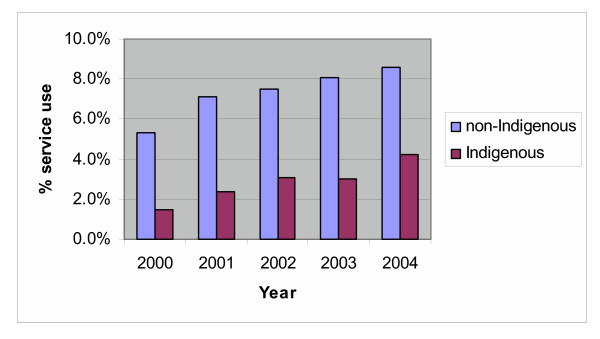
Trends in use of Consulting Room HA items by Indigenous status and Year.

The result of the logistic regression for use of consulting room HA items (see table 2) suggested that Aboriginal and Torres Strait Islander people (3.0%) were significantly less likely to have HAs than the rest of the community (7.4%). There was a significant linear increase in use of the HA items, with use increasing from 5.1% in 2000 to 8.4% in 2004. There was also a significant interaction between Indigenous status and year with use of the HA items increasing slightly more rapidly for Aboriginal and Torres Strait Islander people than the rest of the community (see figure 1). Disparities remained in all years.

Table 3 shows the per cent use of consulting room HA items by State and Territory and Indigenous status. Percentage uptake generally increased with the size of the eligible population with New South Wales (NSW), Queensland (QLD) and Victoria (VIC) having the highest rates and the Northern Territory (NT) having the lowest. In all States and Territories, with the exception of the NT, use was significantly lower among Aboriginal and Torres Strait Islander people. In the NT the pattern was reversed with Aboriginal and Torres Strait Islander people being more likely than the rest of the community to use the HA items. All differences remained significant if it was assumed that Aboriginal and Torres Strait Islander people aged at least 75 years used services at the same rate as the rest of the community.

**Table 3:** Per cent use of Consulting room HA items by Indigenous status and State/Territory

State		75+ yrs non-Indigenous	55+ yrs Indigenous	Total	Difference % (95% CI)	Difference % (95% CI)-dual eligibility adjustment
NSW	Count	156433	1316	157749		
	%	7.7%	2.7%	7.5%	5.0 (5.0–5.1)	4.2 (4.1–4.2)
VIC	Count	111742	566	112308		
	%	7.5%	5.8%	7.5%	2.0 (1.9–2.1)	0.8 (0.7–0.9)
QLD	Count	92970	1359	94329		
	%	9.4%	3.3%	9.1%	6.2 (6.1–6.3)	5.0 (4.9–5.0)
SA	Count	25788	159	25947		
	%	4.8%	1.8%	4.7%	3.0 (2.9–3.1)	2.4 (2.3–2.5)
WA	Count	27620	758	28378		
	%	5.7%	3.4%	5.6%	2.2 (2.4–2.5)	1.6 (1.5–1.7)
TAS	Count	5529	15	5544		
	%	3.6%	.3%	3.5%	3.4 (3.3–3.5)	3.0 (2.9–3.1)
ACT	Count	2496	14	2510		
	%	4.0%	1.8%	4.0%	2.2 (2.1–2.4)	1.8 (1.6–2.0)
NT	Count	197	486	683		
	%	1.9%	2.5%	2.1%	-0.6 (-0.8–-0.3)	-0.9 (-1.1–-0.6)

**Figure 2:**
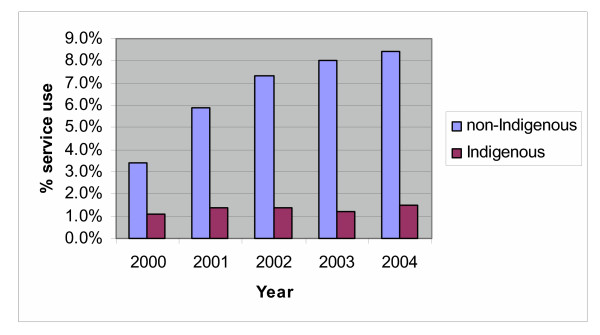
Trends in use of non-Consulting Room HA items by Indigenous status and Year.

The result of the logistic regression for use of non-consulting room HA items (see table 4) suggested that Aboriginal and Torres Strait Islander people (1.3%) were significantly less likely to have HAs than the rest of the community (6.7%). There was a significant linear increase in use of the HA items, with use increasing from 3.4% in 2000 to 8.2% in 2004. There was also a significant interaction between Indigenous status and year with use of the HA items staying stable among Aboriginal and Torres Strait Islander people while increasing in the rest of the community (see figure 2).

**Table 4:** Logistic Regression for use of non-Consulting room HA items by Indigenous status and Year controlling for State/Territory

Variable	Observed service use	Dual eligibility adjustment
	OR (95% CI)	OR (95% CI)
Indigenous status	0.22 (0.21–0.23)	0.34 (0.33–0.35)
Linear trend for year	1.22 (1.21–1.22)	1.22 (1.21–1.22)
Indigenous status * year	0.85 (0.82–0.88)	0.88 (0.86–0.90)

Table 5 shows the per cent use of non-consulting room HA items by State and Territory and Indigenous status. Use of the HA was relatively low in all jurisdictions. Rates of use were much higher in South Australia (SA) and Tasmania (Tas) than in other States and Territories. NT had the lowest take up rate overall. In all States and Territories with the exception of the NT use of non-consulting room HA items was significantly lower among Aboriginal and Torres Strait Islander people. In the NT the trend was reversed with Aboriginal and Torres Strait Islander people being more likely the rest of the community to use the HA items. All differences remained significant when it was assumed that Aboriginal and Torres Strait Islander people aged at least 75 years used services at the same rate as the rest of the community.

**Table 5:** Per cent use of non-Consulting room HA items by Indigenous status and State/Territory

State		75+ yrs non-Indigenous	55+ yrs Indigenous	Total	Difference % (95% CI)	Difference % (95% CI)-dual eligibility adjustment
NSW	Count	132055	658	132713		
	%	6.5%	1.4%	6.4%	5.2 (5.2–5.3)%	4.5 (4.4–4.5)%
VIC	Count	94610	228	94838		
	%	6.4%	2.3%	6.3%	4.2 (4.1–4.2)%	3.2 (3.2–3.3)%
QLD	Count	50911	505	51416		
	%	5.2%	1.2%	5.0%	4.1 (4.0–4.1)%	3.27 (3.2–3.3)%
SA	Count	62203	176	62379		
	%	11.5%	2.0%	11.3%	9.5 (9.4–9.6)%	8.1 (8.0–8.2)%
WA	Count	25725	189	25914		
	%	5.4%	0.8%	5.1%	4.5 (4.5–4.6)%	3.8 (3.7–3.8)%
TAS	Count	15906	29	15935.0		
	%	10.4%	0.5%	10.1%	9.9 (9.8–10.1)%	8.8 (8.7–9.0)%
ACT	Count	2979	4	2983.00		
	%	4.8%	0.5%	4.8%	4.3 (4.1–4.5)%	3.9 (3.7–4.1)%
NT	Count	40	264	304		
	%	0.3%	1.4%	0.9%	-1.0 (-1.2–-0.9)%	-1.0 (-1.2–-0.9)%

### Uptake of Aboriginal and Torres Strait Islander adult HA item compared to uptake of HA items for older people

The logistic regression for the uptake of consulting room HA items in the first three quarters of their introduction suggested that uptake of the HA items for adult Aboriginal and Torres Strait Islander people (710) was lower than for the uptake of the general population item (700) but was higher than uptake of the item for older Aboriginal and Torres Strait Islander people (704, see table 6). Both linear and quadratic trends were significant because rates of use increased substantially after the first quarter and then stabilised in the second and third (see table 7).

**Table 6:** Logistic Regression for uptake of Consulting room HA items in the first 3 quarters after their introduction

Variable	OR (95% CI)
Linear trend-quarters	2.15 (2.02–2.29)
Quadratic trend-quarters	0.63 (0.60–0.66)
75+ yrs Non-Indigenous HA	2.6 (2.49–2.67)
55+ yrs Indigenous HA	0.70 (0.61–0.80)
15–44 yrs Indigenous HA	Reference

**Table 7:** Per cent use of Consulting room HA items in the first 3 quarters after their introduction

HA	Quarter 1	Quarter 2	Quarter 3
	%-95%CI	%-95%CI	%-95%CI
75+ yrs non-Indigenous	0.63 (0.62–0.64)	1.74 (1.73–1.76)	1.73 (1.72–1.75)
55+ yrs Indigenous	0.16 (0.14–0.19)	0.48 (0.44–0.52)	0.51 (0.47–0.55)
15–44 yrs Indigenous	0.23 (0.23–0.24)	0.71 (0.69–0.72)	0.69 (0.67–0.7)

## Discussion

Uptake of HA items was relatively low overall and there was significant and persistent disparity in the uptake of HA items for older people among Aboriginal and Torres Strait Islander people compared to the rest of the community. There were significant differences between the jurisdictions in the overall uptake of items. For consulting room items there appeared to be a relationship between overall State or Territory population and uptake although this was unrelated to other factors such as population density. [16] There was no clear pattern for non-consulting room items. NT was the only jurisdiction where Aboriginal and Torres Strait Islander people used HA items more than non-Aboriginal people. This appeared to occur because of low uptake among non-indigenous Australians rather than higher uptake among Aboriginal and Torres Strait Islander people.

The comparison of the uptake of the HA items in the first three quarters of their use suggested that the uptake of the new adult Aboriginal and Torres Strait Islander item was lower than the uptake of the HA item for older members of the general population. This suggests that additional attention to the causes of barriers to the uptake of HAs among Aboriginal and Torres Strait Islander people may be necessary to achieve the potential benefits associated with these items.

In the evaluation of the EPC program it was suggested that disparities in the uptake of HAs for older people could either be a function of Aboriginal and Torres Strait Islander people having pre-existing care plans or the result of Aboriginal and Torres Strait Islander patients being more likely to see doctors who were ineligible to use Medicare. [2] Differences due to both causes would be expected to decrease over time. Any variation in uptake due to difference in levels of pre-existing HAs would be reduced over time as HAs were renewed. Since the original evaluation an exemption under section 19(2) of the National Health Act has enabled salaried doctors in approved services to bill through Medicare. This has resulted in increased rates of Medicare use at Aboriginal and Torres Strait Islander services. There was some evidence of a slightly faster rate of increase in use among Aboriginal and Torres Strait Islander people for consulting room items though rates appeared stable for non-consulting room items. The persistence of the disparity suggests neither explanation accounts for a large part of the difference in HA uptake between older Aboriginal and Torres Strait Islander people and other Australians.

The EPC evaluation found that awareness of HA items was high among doctors but that lack of awareness of the items among consumers and allied health workers was a barrier to their uptake. [3] Consumer awareness may be particularly important in the use of Aboriginal and Torres Strait Islander items where client identification is an issue. Uptake of HA items was facilitated in practices where practice nurses rather than the doctor undertook the information gathering components. [2] The provision of additional assistance to conduct HAs may be particularly important in Aboriginal and Torres Strait Islander health services where the ratio of walk-in to appointment-based consultations is far higher than in main stream services, making it difficult for doctors to block out the time required for HAs. Even greater barriers may exist in communities were there is no full-time doctor. Others barriers include racism and problems with cross-cultural communication. [10] Barriers associated with cultural appropriateness may be addressed by initiatives such as the development of a guide to conducting health assessments in Aboriginal and Torres Strait islander people. [9] However a multifaceted approach is likely to be required. [2,9]

In any analysis of health services data where clinical data is absent it is difficult to determine appropriate levels of HA use. However it does not seem clinically plausible that Aboriginal and Torres Strait Islander people should be less in need of HAs than comparable other Australians. Ameliorating this situation may require not only further promotion of the items with doctors but further engagement of local primary health infrastructure and the community. [2,5] The evaluation of the HA items and previous initiatives to promote health checks[11] in Aboriginal and Torres Strait islander communities are valuable resources in developing approaches to ensure that the potential health benefit deriving from the new and existing items are delivered.

## Statement of Competing cnterests

The author(s) declare that they have no competing interests.

## Authors' contributions

Margaret Kelaher conceptualised the paper and conducted the analysis. David Dunt, Ian Anderson and David Thomas collaborated in drafting the paper.

**Table 2:** Logistic Regression for use of Consulting room HA items by Indigenous status and Year controlling for State/Territory

Variable	Observed service use	Dual eligibility adjustment
	OR (95% CI)	OR (95% CI)
Indigenous status	0.37 (0.36–0.38)	0.51 (0.50–0.53)
Linear trend for year	1.12 (1.12–1.12)	1.12 (1.12–1.12)
Indigenous status * year	1.11(1.10–1.14)	1.03(1.01–1.06)
